# Progress in salivary glands: Endocrine glands with immune functions

**DOI:** 10.3389/fendo.2023.1061235

**Published:** 2023-02-03

**Authors:** Yu Feng Shang, Yi Yang Shen, Meng Chen Zhang, Min Chao Lv, Tong Ying Wang, Xue Qun Chen, Jun Lin

**Affiliations:** ^1^ Department of Stomatology, Key Laboratory of Oral Biomedical Research of Zhejiang Province, The First Affiliated Hospital, School of Medicine, Zhejiang University, Zhejiang University School of Stomatology, Hangzhou, China; ^2^ National Health Commission and Chinese Academy of Medical Sciences Key Laboratory of Medical Neurobiology, MOE Frontier Science Center for Brain Research and Brain Machine Integration, School of Brain Science and Brain Medicine, Zhejiang University, Hangzhou, China; ^3^ Department of Orthopedics, The Quzhou Affiliated Hospital of Wenzhou Medical University, Quzhou People’s Hospital, Quzhou, China; ^4^ Department of Neurobiology, Department of Neurology of the Second Affiliated Hospital, School of Brain Science and Brain Medicine, Hangzhou, China

**Keywords:** salivary gland, endocrine gland, immune function, saliva, neuroendocrine

## Abstract

The production and secretion of saliva is an essential function of the salivary glands. Saliva is a complicated liquid with different functions, including moistening, digestion, mineralization, lubrication, and mucosal protection. This review focuses on the mechanism and neural regulation of salivary secretion, and saliva is secreted in response to various stimuli, including odor, taste, vision, and mastication. The chemical and physical properties of saliva change dynamically during physiological and pathophysiological processes. Moreover, the central nervous system modulates salivary secretion and function *via* various neurotransmitters and neuroreceptors. Smell, vision, and taste have been investigated for the connection between salivation and brain function. The immune and endocrine functions of the salivary glands have been explored recently. Salivary glands play an essential role in innate and adaptive immunity and protection. Various immune cells such as B cells, T cells, macrophages, and dendritic cells, as well as immunoglobins like IgA and IgG have been found in salivary glands. Evidence supports the synthesis of corticosterone, testosterone, and melatonin in salivary glands. Saliva contains many potential biomarkers derived from epithelial cells, gingival crevicular fluid, and serum. High level of matrix metalloproteinases and cytokines are potential markers for oral carcinoma, infectious disease in the oral cavity, and systemic disease. Further research is required to monitor and predict potential salivary biomarkers for health and disease in clinical practice and precision medicine.

## Introduction

The production and secretion of saliva are the typical functions of the salivary gland (SG). The SG is the first immune barrier in humans and animals, constantly facing external pathogens. Antibodies are known to be present in saliva, and salivary biomarkers are used to diagnose inflammation and antigen challenge, so SGs are considered to be potential immunization sites and useful proxies (1–5). The structure and function of SGs and the properties and composition of saliva, as well as other tissues in the oral cavity are summarized in **Table 1** (1–23). SGs contain a variety of cells, including acinar, epithelial, immune, and endocrine cells. Saliva contains water, ions (Na^+^ and Ca^2+^), immunoglobulins, enzymes, mucins, and hormones. Besides, the more granular convoluted tubules in the submandibular glands produce cell growth factors in mice and rats (**Table 1**) (6).

**Table 1:** Cells, molecules, and microorganisms in oral tissue and saliva.

Tissue		Salivary gland	Saliva	Tonsils	Gingival tissue
Functions		Secretion, Endocrine, Immune	Immune, Regeneration,Digestion, Protection, Mineralization, Lubrication	Immune	Immune, Protection
Cell	Epithelial cell	+	+ (6)	+	+
	Immune cell	+ (1, 3, 4)	+ (6)	+	+
	Neuronal cell	+	–	+	+
	Mesenchymal stem cell	+ (7)	–	–	+
Nuclear acid	DNA	+	+	+	+
	mRNA	+	+	+	+
	miRNA	+	+	+	+
	ncRNA	+	+	+	+
Protein, peptide	Immunoglobin	+ (3–5)	+ (5)	+	+
	Growth factor	+ (8, 9)	+ (10, 11)	-	+ (12)
	Enzyme	+ (13)	+	+	+ (14)
	Functional protein	Mucin, Proline-rich protein, α-amylase, Cystatin, Agglutinin, Glycoprotein;	Albumin, Peroxidase, Mucin, Proline-rich protein, α-amylase, Cystatin, Statherin, Lactoferrin, Defensin;	Defensin (15); Fractalkine (16);	Collagenase (12), Cathepsin (12), Elastase (12), Tryptase (12), Dipeptidyl eptidase (12), Myeloperoxidase (12), Lactate dehydrogenase (12), Lysosome, Lactoferrin, Albumin;
	Hormone	+ (2, 17, 18)	+ (19, 20)	–	+ (21)
	Inorganic substance	NO, Na^+^, K^+^, Cl^–^, HCO_3_^–^, Ca^2+^, NO_3_^–^ (6, 22),	NO, Na^+^, K^+^, Cl^-^, HCO_3_^–^, F^–^, PO_4_^3-^, Ca^2+^, NO_2_^–^ (22), NO_3_^–^ (22), SCN^−^ (23), Mg^2+^ (23)	–	NO, Na^+^, K^+^, F^–^, Ca^2+^, I^–^ (14);
Microorganism	Bacteria	**+ (** 13)	**+ (** 13)	**+**	**+**
	Fungi	**+ (** 13)	**+ (** 13	**-**	**+**
	Viruses	**+ (** 13)	**+ (** 13)	**+**	**+**

+, reported; -, not reported; the numbers of references in brackets or mentioned on maintext.

There are three bilateral salivary glands, the parotid (PG), submandibular (SMG), and sublingual (SLG) gland, as well as thousands of minor SGs in the oral cavity. The PG, located in front of the ear, is the largest SG in humans, but in mice and rats, the SMG is the largest. The SMG lies in the submandibular area and the SLG in the floor of the mouth (6, 24, 25). Stensen’s duct is the main excretory duct of the PG and opens into the oral cavity in the buccal mucosa near the second maxillary molar. The main excretory duct of the SMG is Wharton’s duct, which enters the oral cavity under the tongue by the lingual frenum called the sublingual caruncle. The SLG has small ducts called ducts of Rivinus and a common duct, Bartholin’s duct, which connects with Wharton’s duct at the sublingual caruncle (24, 25).

**Figure 1:**
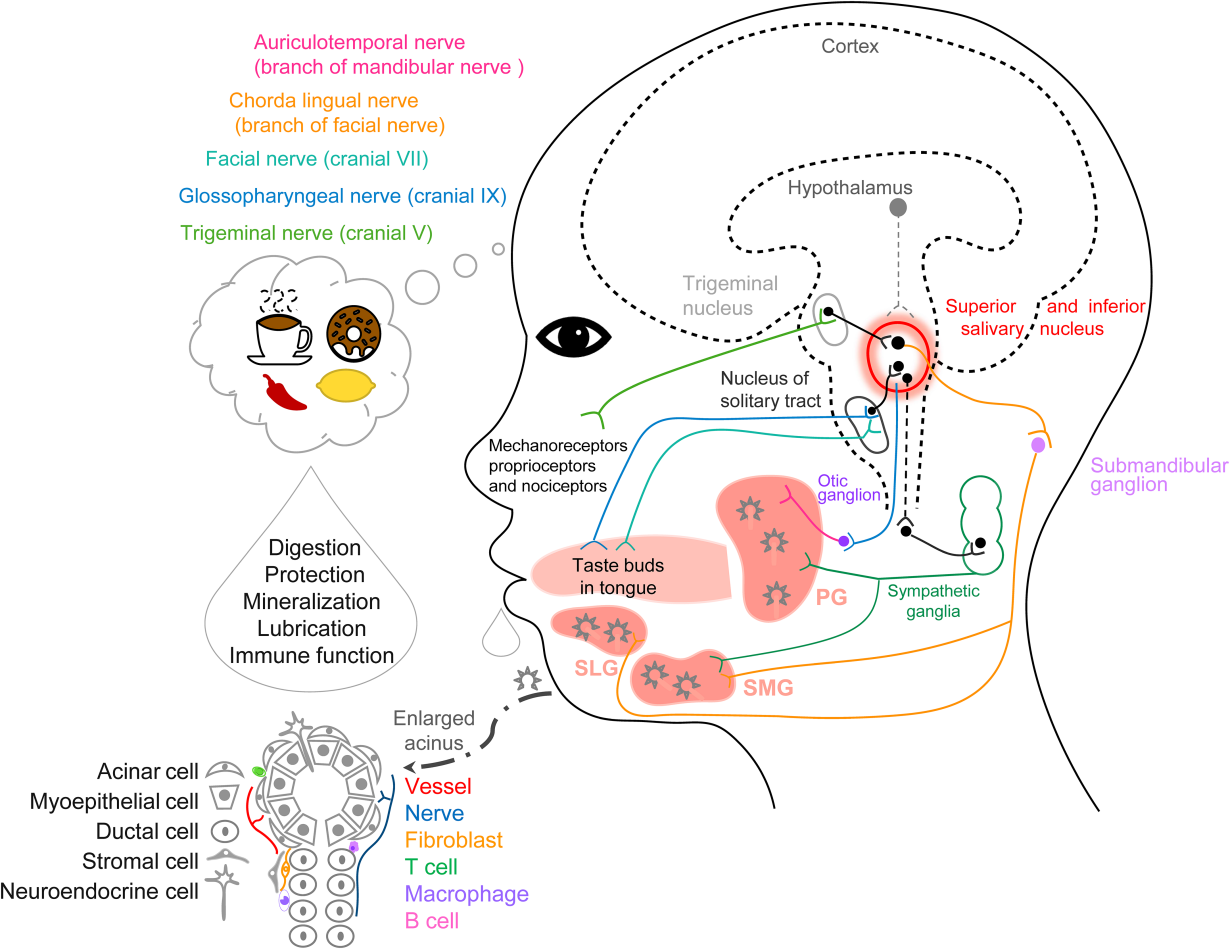
Schematic graph of anatomy, histology, and cell types for the salivary glands and the neurocircuits of saliva secretion.

The SG is composed of parenchyma (glandular secretory tissue) with serous and mucous acinar cells, ductal cells, and myoepithelial cells along with connective tissue. Also, there are fibroblasts, immune cells, neuroendocrine cells, endothelial cells, stromal cells, and pericytes (**Figure 1**) (8, 13). The average daily flow of salivary secretion in adults is 1000–1500 mL/day and >90% is secreted from these three major SGs. Saliva is a fluid that contains water, ions (14, 22, 23, 26), carbohydrates, peptides (enzymes, hormones (9–12, 17–21), and immunoglobulins), exfoliated cells (epithelial and immune cells), nucleic acids, and microorganisms (**Table 1**). Saliva plays important roles in moistening and lubrication, mucosal protection and wound healing, anti-microbial action, tooth protection and immunization (27, 28).

In addition, SGs are involved in neuroendocrine and endocrine functions, taste and smell perception (29, 30). The human minor SG single-cell RNA sequencing atlas (9 samples, 13,824 cells) shows not only the traditional epithelial cells such as acini cells and duct cells, but also diverse immune cells (B lymphocytes) with immunoglobins (such as IgA and IgG), neuroendocrine cells (acidic glycoprotein chromogranin A, CGA and glial cell line-derived neurotrophic factor family receptor α3), and epithelial cells with antibacterial proteins (such as lactoferrin and defensin) (31). Recently, the synthesis and production of corticosterone, testosterone, and melatonin have been reported in the SG of rats, supporting the hypothesis that the SG is an endocrine gland and is involved in the regulation of endocrine function (17, 32).

## Molecular and neural mechanisms of salivary secretion

Salivation is one of the classic Pavlov’s conditioned reflexes *via* environment signals and neuronal mechanisms. The nervous system mediates salivary function, sensory and motor stimuli induce changes in salivary flow and components *via* cellular and molecular mechanisms (8, 33, 34).

Odor induces secretion from SMGs and SLGs but not PGs in humans. The salivary secretion rate increases in response to food or taste-related odors, while different taste-related odors (sweet, savory, and sour) and macronutrient-related odors (carbohydrates, proteins, fats, and low-calorie compounds) induce a similar increase in salivary secretion rate, but do not influence the viscosity, elasticity, ɑ-amylase, and lingual lipase activity. Combinations of stimuli are essential to induce higher flow rates and increase the activity of salivary enzymes to facilitate digestion (35). Human salivary proteome data show that mechanical and gustatory stimuli do not change the total protein concentration, but increases the volume and total amount of protein. It seems that masticatory and gustatory stimuli activate the parasympathetic and sympathetic nervous systems. Both chewing and gustatory stimuli co-regulate the components and concentrations of proteins in human SGs (36). Swallowing efficiency was positively correlated with cerebellar gray matter volume, however, negatively correlated with age in healthy older adults (52-82 years old, 28 female) (37). In clinical study, reconstructing physiological homeostasis of the masticatory complex decrease the thickness of the masseter muscle and increase the height of the maxillary 2^nd^ molar with flattened occlusal curves and curves of Wilson *via* botulinum toxin-A injection, it seems reshaping the masticatory complex can reset the muscle-brain neurocircuits in orthodontics (38).

In humans, tastant recognition signals from the taste buds in the mouth, pharynx, and larynx are transmitted through the facial, glossopharyngeal, and vagus nerves, which terminate in the nucleus of the solitary tract in the brainstem (29, 39). A study designed multisensory food cues (1) odor, (2) odor+vision, (3) odor+vision+taste, and (4) odor+vision+taste+mastication to test the saliva secretion response. The multisensory stimuli result in significant changes in salivary secretion rate and its components depending on the combinations of sensory modalities (40). Transient receptor potential (TRP) ion channels in oral epithelial cells respond to temperature change, irritants (capsaicin), and cooling agents (menthol). Capsaicin (a TRPV1 agonist) also induces a higher sIgA secretion rate with an increase of the β-wave and heart rate variability in the electroencephalogram, TRPV1 increases the sIgA secretion rate through the sympathetic nervous system (41, 42). In another report, TRPV1 agonists (nonivamide, 6.0 × 10^2^ ppm) and TRPM8 agonists (menthol, 1.0 × 10^4^ ppm) also modify the components of saliva and increase protein output from whole mouth saliva. And TRPV1 agonists (nonivamide, 6.0 × 10^2^ ppm) induce an acute salivary cystatin S response, which improves mucosal adhesion. The TRPA1 agonist cinnamaldehyde (1.8 × 10^4^ ppm) is more effective than menthol, but it cannot evoke saliva secretion, suggesting that salivary responses are TRP agonist-specific (43).

Besides, reduced perception of basic taste and smell has been reported in older patients, while there is no significant difference in oral TRP stimulation between older and young groups, indicating that chemo-sensation is retained in the older group. Salivary viscoelasticity decreases with age (higher viscoelasticity in younger *vs* older groups) (44). In humans, chewing induces salivary secretion through the activation of mechanoreceptors in the periodontal ligaments, and proprioceptors and/or nociceptors in the oral mucosa (10).

The salivary flow rate follows a circadian rhythm with the acrophase at ~15:30 and falling to almost zero during sleep. The concentrations of Na^+^, Cl^–^, Ca^2+^, K^+^, and protein in whole saliva also show diverse circadian rhythms in SGs (45). There are rhythmic expression of aquaporin 5 and anoctamin 1 in rat SMG by light condition (46). Since the known clock gene, brain-muscle arnt-like 1 (Bmal1) and period 2(Per2) mRNA expression are expressed in a circadian rhythm pattern in mouse mucous acini and striated ducts, SGs might contain a peripheral clock regulating salivary flow and electrolyte flux (47).

Repeated exposure to an image consistently paired with sour candy immediately increases the saliva secretion rate, but this is not maintained in a second experiment days later (48). In general, food imagination can induce salivary secretion and the mouthwatering sensation (8). In another report, images of foods did not induce an increased salivary flow (49). Increased salivary flow has been found after seeing actual food, and much greater flow occurs when watching other people food. The belief of the participants as to whether they can actually consume these foods has an impact on saliva flow. When the same cookies are colored an unattractive green, the increased salivary flow is eliminated, confirming that humans can develop preferences for palatable food through social learning and emphasizing the importance of palatability (50). Antidepressants and antiparkinsonian medications cause xerostomia because of disturbance in the central nervous system (CNS) (51, 52), this mechanism may be the central accumulation of norepinephrine, which activates α2-adrenoceptors and decreases the activity of parasympathetic salivary neurons in the brainstem (53). Schematic graph summarized the anatomy, histology, and cell types for the salivary glands and the neurocircuits of saliva secretion (**Figure 1**).

The signals from taste-activated chemoreceptors, chewing-activated mechanoreceptors, or nociceptors are transmitted to the nucleus of the solitary tract and then the signals are transmitted to the salivary nuclei (54). Stimulation of the parasympathetic nerves induces protein-poor along with an increased volume of saliva *via* muscarinic cholinergic receptors in SG acinar cells, whereas stimulation of the sympathetic nerves induces protein-rich along with a small volume of saliva *via* β1-adrenoceptors (34, 55, 56). VIP mediates protein secretion and participates in parasympathetic-mediated vasodilatation. VIP-immunoreactive nerve fibers are close to acinar cells, secretory ducts, and blood vessels in human SMGs and PGs. In rat SMGs, VIP evokes acinar degranulation through cAMP-activated protein kinase A (57, 58). Polypeptides in saliva can modulate taste through interacting with the receptors on taste buds. Neuropeptide Y in saliva acts on Y2 receptors expressed in the lingual epithelial cells to induce satiation (59).

## Endocrine and immune functions of salivary glands

SGs produce saliva with endocrine and immune functions, review literature overviewed the resident immune cells (B cells, T cells, macrophages, and dendritic cells) in SGs (60, 61). CgA is stored in the secretory granules of endocrine cells, and it is found in serous and ductal cells in human SMGs through immunohistochemistry (IHC) and *in situ* hybridization (62). In the SMGs of both rats and humans, melatonin and its synthesizing enzyme arylalkylamine N-acetyltransferase have been found in the striated ducts of SMGs; so SGs can produce melatonin (2). In human PGs, SMGs, and labial glands, melatonin is stored in the acinar cells, and released to saliva; heavy immunogold staining has been found in the PG (17). The primary substrate of steroid synthesis and enzyme activity for corticosterone and testosterone production has been detected in the rat SG (32), and estrogen receptor expression has been demonstrated in normal human minor SGs (63). Sex hormone receptors and human epidermal growth factor receptor 2 (HER-2) have been reported in both benign and malignant salivary tumors (64). Androgen receptors and HER-2 are present in high-grade SG carcinomas, and the evaluation of these hormones receptors might benefit targeted therapy or hormone treatment. Steroid hormones are thought to modulate salivary components through autocrine or paracrine pathways (32). Therefore, salivary corticosterone, testosterone, and melatonin might be derived from SGs.

SGs might be one of the critical immune organs involved in innate and adaptive immunity. Vaccines are usually delivered by intramuscular injection, and SGs can be a candidate target for enhancing the immunization response. Immunization of the SG has already been confirmed to protect lethal challenge models of infectious pathogens (65). In the lethal influenza virus infection mouse model, soluble innate inhibitors in saliva such as agglutinin can protect against lower respiratory tract infection. In mice, a potent inhibitor of the virus in saliva can stop the deposited virus in the upper respiratory tract progressing to the lower respiratory tract. Since the saliva in the oral cavity bathes the oropharynx, the potent inhibitor in the saliva can stop the virus in there (66). Since saliva exerts the action of washing away materials such as viruses, a low salivary flow may increase the risk of virus transmission, and a high flow can clean and kill the virus in short time (67, 68).

Saliva functions in innate immunity of the oral cavity may protect against demineralization of teeth. Gel-forming mucins (MUC) are a major constituent, MUC19 is the salivary MUC in mice. A study found MUC19 plays a key role in bacterial clearance, and formation of heterotypic complexes in saliva (binding Streptococcus mutans) in WT- and Mut-*Muc19* mice, this represented a novel innate immune function for mucins. Human MUC19 transcripts in salivary glands (n = 7) and MUC19 glycoproteins in glandular mucous cells and saliva are also found (69). Mucosal immunity including the mucus covering the respiratory tract, surfactants, anti-infectious molecules (sIgA, defensins, and interferons), respiratory epithelial cells, and innate immunity cells (nasal goblet cells and macrophages), form a mucosal barrier that plays a vital role in COVID-19 (70). The mucosal immune system in the nasal and oral cavities, acts as the first barrier, with the appearance of sIgA in the saliva (4). It has been reported that the salivary IgA responding to SARS-CoV-2 persists long-term (3 months) and shows a weak correlation with serum IgA. This suggests that the IgA of the oral cavity comes from the SGs as sIgA (5). IgA is dominant in early virus neutralization. Furthermore, after day 49 post-symptom onset, saliva samples neutralize SARS-CoV-2-pseudotyped viral particles. Anti-receptor binding domain IgA is consistently more abundant in saliva than in serum (71). After a second mRNA vaccine injection, IgG and IgA responses to S-protein and its receptor-binding domain are detectable in human saliva (72). Furthermore, cross-reactive sIgA against SARS-CoV-2 spike 1 is detectable in saliva samples from people without SARS-CoV-2 infection (73). So specific salivary IgA, IgG, and IgM play important roles in airway defense during ongoing SARS-CoV-2 and its prognosis. The decrease in lactoferrin and IgA in COVID-19 patients suggests impairment of the immunoprotective mechanisms of the mucosal barrier; lactoferrin may play an essential role in the pathophysiology of severe cases, since these patients might be vulnerable to secondary airway infection. The concentration of lactoferrin is decreased in patients infected with COVID-19 even after rehabilitation (74). IgA can prevent mucosal infection in the mouth and airway, the lower levels of IgA damage this defense mechanism and promote infection with SARS-CoV-2 and other pathogens during and after infection.

Nasal-spray vaccines not only provoke a whole-body immune response, but also activate a mucosal immune response in the nose and respiratory tract to stop pathogens more quickly (75). Nasal vaccines can make up for the defects of mRNA vaccines in lacking respiratory mucosal immunity, in reaction to the acceleration of viruses evading the immune responses and the increase of transmissibility (76). Intranasal treatment with the novel engineered trimeric ACE2 (eT-ACE2) protein fully protected mice from a δ-variant challenge (500 pfu), and the viral load in lung homogenates was lower 4 days post-infection, while the mortality in the control group was 100% within 8 days in the absence of eT-ACE2 treatment. This study demonstrated that eT-ACE2 (1.5 mg/kg) has a high affinity for S-protein to neutralize SARS-CoV-2 (77). Reducing ACE2 protect from SARS-CoV-2 infection *via* a novel function of farnesoid X receptor, the drug of suppressing this receptor reduced ACE2 mRNA expression in human nasal epithelial cell (78). With regard to mesenchymal stem cell-derived extracellular vesicles from the oral cavity, a review discusses the mechanisms of how this special stem cell can improve neurodegenerative conditions (7). Enteric viruses infect SGs and release into saliva in mice (79), this indicated the viruses infection in SG and saliva as a potential risk transmission route through talking, coughing, sneezing and kissing, not just fecal contamination. The new study from healthcare workers reported the mRNA-BNT162b2 vaccination drastically induced a systemic immune response by boosting neutralizing antibodies in serum, but not in saliva, therefore oral mucosal immunity is not activated and cannot stop virus acquisition *via* this route (80). Mosquito (*Aedes aegypti*) salivary gland extract is known to regulate host immune responses and pathogen transmission, a salivary protein (LTRIN) have been identified from mosquito, this protein can facilitates the transmission of Zika virus by lymphotoxin-β receptor and interfering, LTRIN might a target for Zika infection and treatment (81). In mosquito saliva, anticoagulant, vasodilatory, and immunomodulatory activities is detectable after the bite, the review discussed the innate and adaptive response in skin to mosquito saliva (82).

## Dysfunction and disorder in the salivary gland

Systemic diseases, such as infection, inflammatory disorders, genetic diseases, and neoplastic diseases and disorders impact the function of oral organs (83–85). There are many reviews of salivary function and secretion in health and disease (10, 86). Hyposalivation is the objective reduction of salivary secretion, Xerostomia is the feeling of oral dryness and a complaint in hyposalivation. Sjögren syndrome (SS) is a well-known autoimmune disease, in which the exocrine glands are primarily affected by oral dryness, and autoimmune epithelitis is its major pathogenesis. Epithelial cells act as the central regulators of autoimmune responses through presenting antigens. The accumulation of immune cells regulates the local immune responses and further activates epithelial cells leading to a vicious cycle of epithelial cell and immune cell interaction. The epithelium of the SGs is impaired by abnormal B cell and T cell infiltration and secondary chronic inflammation causes the loss of physiological functions (87). IL-17 and chemokine receptor 9^+^/α4β7^-^ Th17 cells promoted the inflammation, dysfunction, and cell death in salivary gland of NOD/ShiLtJ mice, in human SS patient, serum IgA and IL-17 level are higher with lower retinoic acid level, and there are a numbers of chemokine receptor 9 and IL-17 double-positive cells in biopsy specimens of salivary gland (88). A test from the minor salivary glands of SS patients and healthy individuals, there is no significant difference in leptin expression and leptin receptor distribution in SG, therefore leptin seems not association with the pathogenesis of SS (18). In patients with plaque psoriasis, a chronic, immune-related disease, the secretory function of the PG and SMG is lost and salivary amylase activity and total protein concentration is decreased, meanwhile TNF-1, IL-2, and INF-γ level are higher with lower IL-10 (89), salivary total oxidant status and oxidative status index may be potential diagnostic biomarkers for plaque psoriasis (90).

Angiotensin-converting enzyme 2 (ACE2) and transmembrane protease serine 2 (TMPRSS2) are target molecules for SARS-CoV-2. ACE2 is the only human cellular receptor, furthermore, TMPRSS2 cleavage of the viral spike protein and ACE2 begin the host-pathogen interaction process (91, 92). In saliva, furin cleaves the S protein into S1 and S2 domains and is associated with the infectivity of SARS-CoV-2 (93). S1 with the ACE2 recognition motif is responsible for binding to the cell. The S2 domain regulates fusion of the virus with the host cell (94). So the co-expression of ACE2, TMPRSS2, and furin in the SGs is pivotal for SARS-CoV-2 to change the function of salivary secretion, IHC analyses have shown these three proteins are expressed in the SMGs of humans and rats (95). In SG sections from adult patients with benign disorders (sialolithiasis and mucocele), ACE2 and TMPRSS2 expression is present in the cytoplasm of serous acinar cells (96). Moreover, when samples of SGs from fatal COVID-19 cases are tested using qRT-PCR, IHC, electron microscopy, and histopathological analysis, infection and replication of COVID-19 are found, as shown by the presence of ACE2 and TMPRSS receptors (97). The interaction between ACE2 and the virus downregulates the receptor and induces the accumulation of angiotensin II (98), which reduces parotid secretion through vasoconstrictor action and changes in water and electrolyte transport (99). Losartan (an angiotensin II receptor blocker) decreases saliva and total protein secretion, on account of reducing the mRNA expression of the renin-angiotensin system (RAS) in the parotid and the microthrombotic occlusion in vessels of the SGs (100). The downregulation of ACE2 leads to a dysregulated RAS, inflammation, and constant damage throughout the duration of COVID-19. The plasma ACE2 activity is elevated in patients even after COVID-19 infection for 114 days (101). SARS-CoV-2 can bind to ACE2 receptors in SGs and lyses the cells to induce acute sialadenitis. Although fibrous repair and hyperplasia can repair the damage, the consequence is SG hyposecretion and stenosis in the ducts (102).

Aging is a physiological process with dry mouth and lower salivary flow rates. Fat tissue is increased in SGs from the aged population, and the proportional volume of acinar cell secretion is reduced in elderly individuals (103). Notably, the reduction in the number of olfactory and taste receptors leads to the diminished intensity of stimulation and a decrease in the blood perfusion at the glandular level in elderly individuals (103), supporting the idea that the CNS is involved in the functions of SGs.

Medication-induced salivary dysfunction is a common cause of xerostomia; the drugs target various receptors in SGs and the CNS, hyposalivation, alteration of chemical composition and physical properties, as well as cognitive functions are the causes of xerostomia (60, 104, 105). SG damage includes radiation-induced loss of acinar cells, impaired parasympathetic innervation, and damaged vascular structures (106). Atrophy of the SGs occurs secondary to their denervation in patients (107). The weight of SMGs and aquaporin 5 expression in SMGs are decreased after parasympathectomy (108). Chronic denervation of the trigeminal nerve is linked to atrophy, and increased fatty tissue in the dog PG has been reported, trigeminal dysfunction results in accumulation of abnormal saliva and decreased weight and size of the ipsilateral SG. Denervation and loss of the masticatory–salivary reflex is one of the reasons for the atrophy of SGs (109).

Endocrine diseases such as diabetes mellitus are associated with salivary gland dysfunction in humans (110), polyuria and dehydration cause reduced salivary flow. Enlarged acinar cells and ductal atrophy occur in the SMG, and salivary flow is reduced in mice with type-2 diabetes along with increased mitochondrial dysfunction and higher expression of PTEN-induced putative kinase 1 and parkin. This suggests a mitophagy mechanism in hyposalivation (111). Patients with SG dysfunction often complain about difficulty in swallowing, chewing, and speaking (51) as well as manifestations of halitosis (112), dry buccal mucosa, glossitis (113), cracked and peeling lips (114), oral candidiasis (115, 116), and active caries (117). Moreover, salivary dysfunction can have severe consequences, including appetite loss, sleep deprivation, depression, and impaired immune function. Saliva and SGs are critical for taste sensation: dissolved tastants in saliva act on the taste receptors on taste buds to produce sensation (30). Hyposalivation decreases the tastants released from foods, resulting in changed taste. Xerostomia is one of the severe consequences in patients undergoing chemotherapy (118), so it is important to assess the changes in taste and smell to guide the dosage of drug in real time, rather than computed tomography or magnetic resonance imaging. Salivary hypofunction in the elderly is associated with less food consumption. Hyposalivation impairs chewing and swallowing, resulting in the loss of appetite and an imbalance of food intake, since flavor perception contributes to food intake (119, 120). Hyposalivation in older adults along with lower nutrient intake causes further health problems (121). Besides, hyposalivation leads to insomnia, fragmented sleep, and daytime somnolence, because of drinking at night and disruption of the circadian rhythm. In SS patients, poor sleep quality results in depression and impaired immune function (122, 123). Given the deficiency of anti-viral proteins in saliva and of the oral and respiratory mucosal barrier, hyposalivation is a potential risk factor for SARAS-CoV-2 and other respiratory infections (124, 125).

## Salivary biomarkers in health and diseases

Salivary biomarkers are a powerful tool for prognosis, diagnosis, and precision medicine. Increasingly, discoveries in the study of saliva and meta-analysis by clinical scientists support the conclusion that salivary biomarkers can act as a reporter for physiological and pathophysiological condition, as well as the local oral and periodontal environment. This has been applied to early detection and monitoring in health and disease (126–128).

Increases in matrix metalloproteinase (MMP)-8, MMP-9, tissue inhibitor of matrix metalloproteinase-1, myeloperoxidase, and complement component C3c in saliva are strongly correlated with periodontitis (129). The study reported increased S100A12 expression in inflamed gingival tissue and involved in periodontitis (130). And high baseline levels of complement component C3c in saliva can serve as a marker for evaluating responses to full-mouth non-surgical periodontal treatment, including oral hygiene instructions, scaling, and root planing (131).

SS is characterized by lymphoplasmacytic infiltration of the salivary and lacrimal glands. In the salivary proteome of SS patients, fatty acid-binding protein, β-actin, and glutathione S-transferase are decreased, while α-amylase precursor, cystatin precursor, and keratin6-L are increased (132). Another study reported that the levels of cystatin C, lysozyme C, and carbonic anhydrase VI are lower, while the levels of psoriasin and caspase 14 are higher in SS patients (133).

COVID-19, the infectious disease caused by SARS-CoV-2 (one of the coronaviruses), was declared a pandemic by the World Health Organization and remains at a steady state (134). Salivary testing and monitoring might provide an easy and effective point-of-care platform for the quick and long-term diagnosis of COVID-19 (135). Eleven cases out of 12 (91.67%) and 20 out of 23 (86.96%) COVID-19 patients are reported to be 2019-nCoV RNA-positive in saliva (136, 137). However, in saliva swabs, half of 15 COVID-19 patients and 13 cases out of 31 (41.94%) are 2019-nCoV RNA-positive in saliva (135, 138). The diagnostic value of saliva greatly depends on how it is obtained. In samples from salivary gland ducts, 4 of 31 COVID-19 patients (12.90%) are found to be 2019-nCoV RNA-positive (135).

Saliva is a sensitive and specific prognostic and diagnostic tool by measuring the concentration of free circulating cortisol and melatonin (139, 140). It is also used as a conventional test in clinical and basic research. Salivary samples permit the real-time diagnosis and monitoring of hormonal secretions and the control of the concentration of hormones used as drugs (such as glucocorticoid replacement therapy) (141). In 100 healthy volunteers, there are different patterns in 3 biomarkers (cortisol, amylase and CGA) on day- and night-saliva: higher level cortisol in lower BMI (body mass index) group, and higher CGA with lower amylase in older group, and lower CGA in male group (*vs* female); the cortisol levels follow the higher level on day with lower on night as previous studies (142). Salivary cortisol is useful for the screening of Cushing’s syndrome, it is easy to detect the impaired circadian rhythm of the hypothalamic–pituitary–adrenal axis presented by Cushing’s syndrome patients by the higher cortisol values in saliva at night (143, 144). Inflammatory profiles of saliva and serum in inflammatory bowel disease share similar elevated IL-6 and MMP-10 levels in stimulated saliva (145). Macrophage colony-stimulating factor 1 in saliva is positively associated with caries in healthy children (7–9 years old) (146).

Several cytokines in saliva have been regarded as possible biomarkers for oral squamous cell carcinoma (OSCC). Salivary IL-6 levels are elevated not only in OSCC patients but also in chronic oral inflammatory diseases such as chronic periodontitis (CP) and oral lichen planus (OLP). The salivary IL-6 level in OSCC patients is significantly higher than that of in healthy volunteers, CP, and OLP patients, indicating that IL-6 can serve as a promising biomarker for OSCC detection, and can also be biomarker for identifying CP and/or OLP (147, 148). The salivary IL-8 level is also elevated in both OSCC patients and oral pre-cancer patients compared to healthy controls, so it might be candidate for the risk of oral diseases (149).

A salivary microRNA has been reported as a new biomarker for OSCC screening. miR-30c-5p is significantly decreased in the saliva of OSCC patients. High expression of the miR-targeted genes is negatively correlated with miR-30c-5p in tissue, and this might be linked to the short overall survival in patients (150).

In HIV/SIV-induced periodontal disease in the rhesus macaques model, phytocannabinoids reduce gingival/systemic inflammation and salivary dysbiosis and improve the metabolic dysfunction (151). In children with chronic kidney disease, the higher activity of peroxidase and superoxide dismutase in stimulated saliva, as well as elevated concentrations of uric acid and albumin in non-stimulated saliva and stimulated saliva have been reported (*vs* control). Salivary advanced oxidation protein products can be a potential biomarker and diagnostic value in children patients. Additionally, ferric ion reducing antioxidant and uric acid in salivary are higher in children patients, it might be as marker for disease progression (152, 153). Salivary biomarkers will be effective and non-invasive in the prognosis and monitoring of disease progression and evaluating the response to treatment.

## Conclusions and perspectives

In this review, we summarize the progress in studies of salivary secretion and function. Many sensory stimuli can modulate the salivary secretion, flow rate, and composition *via* the CNS. Substantial evidence supports the endocrine and immune functions of SGs. Abundant biomarkers in saliva are a potential tool for monitoring, prognosis, diagnosis, and precision medicine in health and disease. Based on endocrine and neuroendocrine reflexes in salivary secretion, it is necessary to explore the neural circuits in the brain–peripheral organ axis.

## Author contributions

JL and XC supervised the project. YS, JL and XC wrote drafts of the manuscript. MCZ, YS, ML and TW collected the literature. All authors contributed to the article and approved the submitted version.

## References

[1] Liu G Zhang F Wang R London SD London L. Salivary gland immunization *via* wharton's duct activates differential T-cell responses within the salivary gland immune system. FASEB J (2019) 33(5):6011–22. doi: 10.1096/fj.201801993R PMC6463922 30817215

[2] Shimozuma M Tokuyama R Tatehara S Umeki H Ide S Mishima K. Expression and cellular localizaion of melatonin-synthesizing enzymes in rat and human salivary glands. Histochem Cell Biol (2011) 135(4):389–96. doi: 10.1007/s00418-011-0800-8 21437622

[3] Ponzio TA Sanders JW. The salivary gland as a target for enhancing immunization response. Trop Dis Travel Med Vaccines (2017) 3:4. doi: 10.1186/s40794-017-0047-z 28883974 PMC5531011

[4] Russell MW Moldoveanu Z Ogra PL Mestecky J. Mucosal immunity in COVID-19: a neglected but critical aspect of SARS-CoV-2 infection. Front Immunol (2020) 11:611337. doi: 10.3389/fimmu.2020.611337 33329607 PMC7733922

[5] Isho B Abe KT Zuo M Jamal AJ Rathod B Wang JH. Persistence of serum and saliva antibody responses to SARS-CoV-2 spike antigens in COVID-19 patients. Sci Immunol (2020) 5(52):eabe5511. doi: 10.1126/sciimmunol.abe5511 33033173 PMC8050884

[6] Amano O Mizobe K Bando Y Sakiyama K. Anatomy and histology of rodent and human major salivary glands: -overview of the Japan salivary gland society-sponsored workshop. Acta Histochem Cytochem (2012) 45(5):241–50. doi: 10.1267/ahc.12013 PMC3496860 23209333

[7] Pourhadi M Zali H Ghasemi R Vafaei-Nezhad S. Promising role of oral cavity mesenchymal stem cell-derived extracellular vesicles in neurodegenerative diseases. Mol Neurobiol (2022) 59(10):6125–40. doi: 10.1007/s12035-022-02951-y 35867205

[8] Proctor GB. The physiology of salivary secretion. Periodontology 2000 (2016) 70(1):11–25. doi: 10.1111/prd.12116 26662479

[9] Li SS Wu CZ Zhang BW Qiu L Chen W Yuan YH. Nerve growth factor protects salivary glands from irradiation-induced damage. Life Sci (2021) 265:118748. doi: 10.1016/j.lfs.2020.118748 33189827

[10] Pedersen AML Sorensen CE Proctor GB Carpenter GH Ekstrom J. Salivary secretion in health and disease. J Oral Rehabil (2018) 45(9):730–46. doi: 10.1111/joor.12664 29878444

[11] Blochowiak K Sokalski J Golusinska E Trzybulska D Witmanowski H Bodnar M. Salivary levels and immunohistochemical expression of selected angiogenic factors in benign and malignant parotid gland tumours. Clin Oral Investig (2019) 23(3):995–1006. doi: 10.1007/s00784-018-2524-9 29926253

[12] Rakmanee T Calciolari E Olsen I Darbar U Griffiths GS Petrie A. Expression of growth mediators in the gingival crevicular fluid of patients with aggressive periodontitis undergoing periodontal surgery. Clin Oral Invest (2019) 23(8):3307–18. doi: 10.1007/s00784-018-2752-z 30498980

[13] Porcheri C Mitsiadis TA. Physiology, pathology and regeneration of salivary glands. Cells (2019) 8(9):976. doi: 10.3390/cells8090976 31455013 PMC6769486

[14] Subbarao KC Nattuthurai GS Sundararajan SK Sujith I Joseph J Syedshah YP. Gingival crevicular fluid: an overview. J Pharm Bioallied Sci (2019) 11(Suppl 2):S135–S9. doi: 10.4103/JPBS.JPBS_56_19 PMC6555362 31198325

[15] Kamekura R Imai R Takano K Yamashita K Jitsukawa S Nagaya T. Expression and localization of human defensins in palatine tonsils. Adv Otorhinolaryngol (2016) 77:112–8. doi: 10.1159/000441888 27115892

[16] Koclu Hetemoglu E Turkoglu Babakurban S Terzi YK Sahin FI Erbek SS. The differences in the expression of fractalkine and its receptor in conditions of tonsillar hypertrophy and chronic tonsillitis. Auris Nasus Larynx (2019) 46(4):565–9. doi: 10.1016/j.anl.2018.12.001 30554983

[17] Isola M Lilliu MA. Melatonin localization in human salivary glands. J Oral Pathol Med (2016) 45(7):510–5. doi: 10.1111/jop.12409 26694219

[18] Erbasan F Alikanoglu AS Yazisiz V Karasu U Balkarli A Sezer C. Leptin and leptin receptors in salivary glands of primary sjogren's syndrome. Pathol Res Pract (2016) 212(11):1010–4. doi: 10.1016/j.prp.2016.08.009 27688083

[19] Umeki H Tokuyama R Ide S Okubo M Tadokoro S Tezuka M. Leptin promotes wound healing in the oral mucosa. PloS One (2014) 9(7):e101984. doi: 10.1371/journal.pone.0101984 25033454 PMC4102470

[20] Ngamchuea K Chaisiwamongkhol K Batchelor-McAuley C Compton RG. Chemical analysis in saliva and the search for salivary biomarkers - a tutorial review. Analyst (2017) 143(1):81–99. doi: 10.1039/c7an01571b 29149225

[21] Sar SK Shetty D Kumar P Juneja S Sharma P. Leptin levels in gingival crevicular fluid during canine retraction: *in vivo* comparative study. J Orthod (2019) 46(1):27–33. doi: 10.1177/1465312518820533 31056072

[22] Ma L Hu L Feng X Wang S. Nitrate and nitrite in health and disease. Aging Dis (2018) 9(5):938–45. doi: 10.14336/AD.2017.1207 PMC6147587 30271668

[23] Roblegg E Coughran A Sirjani D. Saliva: An all-rounder of our body. Eur J Pharm Biopharm (2019) 142:133–41. doi: 10.1016/j.ejpb.2019.06.016 31220573

[24] Atkinson C Fuller J Huang B. Cross-sectional imaging techniques and normal anatomy of the salivary glands. Neuroimaging Clinics North America (2018) 28(2):137–58. doi: 10.1016/j.nic.2018.01.001 29622110

[25] Holmberg KV Hoffman MP. Anatomy, biogenesis and regeneration of salivary glands. Monogr Oral Sci (2014) 24:1–13. doi: 10.1159/000358776 24862590 PMC4048853

[26] Chen M Cai W Zhao S Shi L Chen Y Li X. Oxidative stress-related biomarkers in saliva and gingival crevicular fluid associated with chronic periodontitis: A systematic review and meta-analysis. J Clin Periodontol (2019) 46(6):608–22. doi: 10.1111/jcpe.13112 30989678

[27] Rodrigues Neves C Buskermolen J Roffel S Waaijman T Thon M Veerman E. Human saliva stimulates skin and oral wound healing. vitro. J Tissue Eng Regener Med (2019) 13(6):1079–92. doi: 10.1002/term.2865 PMC6593997 30968584

[28] Carpenter GH. The secretion, components, and properties of saliva. Annu Rev Food Sci Technol (2013) 4:267–76. doi: 10.1146/annurev-food-030212-182700 23464573

[29] Vincis R Fontanini A. Central taste anatomy and physiology. Handb Clin Neurol (2019) 164:187–204. doi: 10.1016/B978-0-444-63855-7.00012-5 31604547 PMC6989094

[30] Dawes C Pedersen AM Villa A Ekstrom J Proctor GB Vissink A. The functions of human saliva: A review sponsored by the world workshop on oral medicine VI. Arch Oral Biol (2015) 60(6):863–74. doi: 10.1016/j.archoralbio.2015.03.004 25841068

[31] Huang N Perez P Kato T Mikami Y Okuda K Gilmore RC. SARS-CoV-2 infection of the oral cavity and saliva. Nat Med (2021) 27(5):892–903. doi: 10.1038/s41591-021-01296-8 33767405 PMC8240394

[32] Ieko T Sasaki H Maeda N Fujiki J Iwano H Yokota H. Analysis of corticosterone and testosterone synthesis in rat salivary gland homogenates. Front Endocrinol (Lausanne) (2019) 10:479. doi: 10.3389/fendo.2019.00479 31379745 PMC6650613

[33] Proctor GB Carpenter GH. Salivary secretion: mechanism and neural regulation. Monogr Oral Sci (2014) 24:14–29. doi: 10.1159/000358781 24862591

[34] Proctor GB Carpenter GH. Regulation of salivary gland function by autonomic nerves. Auton Neurosci (2007) 133(1):3–18. doi: 10.1016/j.autneu.2006.10.006 17157080

[35] Morquecho-Campos P Bikker FJ Nazmi K de Graaf K Laine ML Boesveldt S. Impact of food odors signaling specific taste qualities and macronutrient content on saliva secretion and composition. Appetite (2019) 143:104399. doi: 10.1016/j.appet.2019.104399 31401237

[36] Carreira L Castelo PM Simoes C Silva FCE Viegas C Lamy E. Changes in salivary proteome in response to bread odour. Nutrients (2020) 12(4):1002. doi: 10.3390/nu12041002 32260553 PMC7230670

[37] Lin CS Wu CY Wang DH Lin HH Lo WL. Brain signatures associated with swallowing efficiency in older people. Exp Gerontol (2019) 115:1–. doi: 10.1016/j.exger.2018.11.007 30415067

[38] Li X Feng X Li J Bao X Xu J Lin J. Can botulinum toxin-A contribute to reconstructing the physiological homeostasis of the masticatory complex in short-faced patients during occlusal therapy? A prospective pilot study. Toxins (Basel) (2022) 14(6):374. doi: 10.3390/toxins14060374 35737035 PMC9227267

[39] Bradley RM Fukami H Suwabe T. Neurobiology of the gustatory-salivary reflex. Chem Senses (2005) 30(Suppl 1):i70–1. doi: 10.1093/chemse/bjh118 15738201

[40] Morquecho-Campos P Bikker FJ Nazmi K de Graaf K Laine ML Boesveldt S. A stepwise approach investigating salivary responses upon multisensory food cues. Physiol Behav (2020) 226:113116. doi: 10.1016/j.physbeh.2020.113116 32750433

[41] Wang B Danjo A Kajiya H Okabe K Kido MA. Oral epithelial cells are activated *via* TRP channels. J Dent Res (2011) 90(2):163–7. doi: 10.1177/0022034510385459 21149857

[42] Kono Y Kubota A Taira M Katsuyama N Sugimoto K. Effects of oral stimulation with capsaicin on salivary secretion and neural activities in the autonomic system and the brain. J Dent Sci (2018) 13(2):116–23. doi: 10.1016/j.jds.2017.08.007 PMC6388832 30895106

[43] Houghton JW Carpenter G Hans J Pesaro M Lynham S Proctor G. Agonists of orally expressed TRP channels stimulate salivary secretion and modify the salivary proteome. Mol Cell Proteomics (2020) 19(10):1664–76. doi: 10.1074/mcp.RA120.002174 PMC8014997 32651226

[44] Pushpass RG Daly B Kelly C Proctor G Carpenter GH. Altered salivary flow, protein composition, and rheology following taste and TRP stimulation in older adults. Front Physiol (2019) 10:652. doi: 10.3389/fphys.2019.00652 31214042 PMC6555201

[45] Wang Z Shen MM Liu XJ Si Y Yu GY. Characteristics of the saliva flow rates of minor salivary glands in healthy people. Arch Oral Biol (2015) 60(3):385–92. doi: 10.1016/j.archoralbio.2014.11.016 25526622

[46] Satou R Shibukawa Y Kimura M Sugihara N. Light conditions affect rhythmic expression of aquaporin 5 and anoctamin 1 in rat submandibular glands. Heliyon (2019) 5(11):e02792. doi: 10.1016/j.heliyon.2019.e02792 31844723 PMC6895735

[47] Zheng L Seon YJ McHugh J Papagerakis S Papagerakis P. Clock genes show circadian rhythms in salivary glands. J Dent Res (2012) 91(8):783–8. doi: 10.1177/0022034512451450 PMC3398790 22699207

[48] Kershaw JC Running CA. Conditioning of human salivary flow using a visual cue for sour candy. Arch Oral Biol (2018) 92:90–5. doi: 10.1016/j.archoralbio.2018.05.010 29778624

[49] Ilangakoon Y Carpenter GH. Is the mouthwatering sensation a true salivary reflex? J Texture Stud (2011) 42(3):212–6. doi: 10.1111/j.1745-4603.2011.00290.x

[50] Spence C. Mouth-watering: The influence of environmental and cognitive factors on salivation and gustatory/flavor perception. J Texture Stud (2011) 42(2):157–71. doi: 10.1111/j.1745-4603.2011.00299.x

[51] Millsop JW Wang EA Fazel N. Etiology, evaluation, and management of xerostomia. Clin Dermatol (2017) 35(5):468–76. doi: 10.1016/j.clindermatol.2017.06.010 28916028

[52] Johnsson M Winder M Zawia H Lodoen I Tobin G Gotrick B. *In vivo* studies of effects of antidepressants on parotid salivary secretion in the rat. Arch Oral Biol (2016) 67:54–60. doi: 10.1016/j.archoralbio.2016.03.010 27023402

[53] Cappetta K Beyer C Johnson JA Bloch MH. Meta-analysis: risk of dry mouth with second generation antidepressants. Prog Neuropsychopharmacol Biol Psychiatry (2018) 84(Pt A):282–93. doi: 10.1016/j.pnpbp.2017.12.012 29274375

[54] Pedersen A Sorensen CE Proctor GB Carpenter GH. Salivary functions in mastication, taste and textural perception, swallowing and initial digestion. Oral Dis (2018) 24(8):1399–416. doi: 10.1111/odi.12867 29645367

[55] Nakamura T Matsui M Uchida K Futatsugi A Kusakawa S Matsumoto N. M(3) muscarinic acetylcholine receptor plays a critical role in parasympathetic control of salivation in mice. J Physiol (2004) 558(Pt 2):561–75. doi: 10.1113/jphysiol.2004.064626 PMC1664962 15146045

[56] Gautam D Heard TS Cui Y Miller G Bloodworth L Wess J. Cholinergic stimulation of salivary secretion studied with M1 and M3 muscarinic receptor single- and double-knockout mice. Mol Pharmacol (2004) 66(2):260–7. doi: 10.1124/mol.66.2.260 15266016

[57] Del Fiacco M Quartu M Ekstrom J Melis T Boi M Isola M. Effect of the neuropeptides vasoactive intestinal peptide, peptide histidine methionine and substance p on human major salivary gland secretion. Oral Dis (2015) 21(2):216–23. doi: 10.1111/odi.12249 24725136

[58] Culp DJ Zhang Z Evans RL. VIP And muscarinic synergistic mucin secretion by salivary mucous cells is mediated by enhanced PKC activity *via* VIP-induced release of an intracellular Ca^2+^ pool. Pflugers Arch (2020) 472(3):385–403. doi: 10.1007/s00424-020-02348-7 31932898 PMC7058512

[59] Fabian TK Beck A Fejerdy P Hermann P Fabian G. Molecular mechanisms of taste recognition: considerations about the role of saliva. Int J Mol Sci (2015) 16(3):5945–74. doi: 10.3390/ijms16035945 PMC4394514 25782158

[60] Chibly AM Aure MH Patel VN Hoffman MP. Salivary gland function, development, and regeneration. Physiol Rev (2022) 102(3):1495–552. doi: 10.1152/physrev.00015.2021 PMC9126227 35343828

[61] Lu L Tanaka Y Ishii N Sasano T Sugawara S. CD103^+^CD11b^–^ salivary gland dendritic cells have antigen cross-presenting capacity. Eur J Immunol (2017) 47(2):305–13. doi: 10.1002/eji.201646631 27861804

[62] Saruta J Tsukinoki K Sasaguri K Ishii H Yasuda M Osamura YR. Expression and localization of chromogranin a gene and protein in human submandibular gland. Cells Tissues Organs (2005) 180(4):237–44. doi: 10.1159/000088939 16330879

[63] Tsinti M Kassi E Korkolopoulou P Kapsogeorgou E Moutsatsou P Patsouris E. Functional estrogen receptors alpha and beta are expressed in normal human salivary gland epithelium and apparently mediate immunomodulatory effects. Eur J Oral Sci (2009) 117(5):498–505. doi: 10.1111/j.1600-0722.2009.00659.x 19758244

[64] Can NT Lingen MW Mashek H McElherne J Briese R Fitzpatrick C. Expression of hormone receptors and HER-2 in benign and malignant salivary gland tumors. Head Neck Pathol (2018) 12(1):95–104. doi: 10.1007/s12105-017-0833-y 28681314 PMC5873488

[65] Ponzio TA Sanders JW. The salivary gland as a target for enhancing immunization response. Trop diseases travel Med Vaccines (2017) 3:4–. doi: 10.1186/s40794-017-0047-z PMC5531011 28883974

[66] Ivinson K Deliyannis G McNabb L Grollo L Gilbertson B Jackson D. Salivary blockade protects the lower respiratory tract of mice from lethal influenza virus infection. J Virol (2017) 91(14):e00624-17. doi: 10.1128/JVI.00624-17 28446669 PMC5487578

[67] Li Y Ren B Peng X Hu T Li J Gong T. Saliva is a non-negligible factor in the spread of COVID-19. Mol Oral Microbiol (2020) 35(4):141–5. doi: 10.1111/omi.12289 PMC7267240 32367576

[68] Tada A Senpuku H. The impact of oral health on respiratory viral infection. Dent J (Basel) (2021) 9(4):43. doi: 10.3390/dj9040043 33924596 PMC8069613

[69] Culp DJ Robinson B Cash MN Bhattacharyya I Stewart C Cuadra-Saenz G. Salivary mucin 19 glycoproteins: innate immune functions in streptococcus mutans-induced caries in mice and evidence for expression in human saliva. J Biol Chem (2015) 290(5):2993–3008. doi: 10.1074/jbc.M114.597906 25512380 PMC4317041

[70] Tosta E. The seven constitutive respiratory defense barriers against SARS-CoV-2 infection. Rev Soc Bras Med Trop (2021) 54:e04612021. doi: 10.1590/0037-8682-0461-2021 34932765 PMC8687496

[71] Sterlin D Mathian A Miyara M Mohr A Anna F Claër L. IgA dominates the early neutralizing antibody response to SARS-CoV-2. Sci Transl Med (2021) 13(577):eabd2223. doi: 10.1126/scitranslmed.abd2223 33288662 PMC7857408

[72] Ketas TJ Chaturbhuj D Portillo VMC Francomano E Golden E Chandrasekhar S. Antibody responses to SARS-CoV-2 mRNA vaccines are detectable in saliva. Pathog Immun (2021) 6(1):116–34. doi: 10.20411/pai.v6i1.441 PMC8201795 34136730

[73] Tsukinoki K Yamamoto T Handa K Iwamiya M Saruta J Ino S. Detection of cross-reactive immunoglobulin a against the severe acute respiratory syndrome-coronavirus-2 spike 1 subunit in saliva. PloS One (2021) 16(11):e0249979. doi: 10.1371/journal.pone.0249979 34813596 PMC8610234

[74] Santos JGO Migueis DP Amaral JBD Bachi ALL Boggi AC Thamboo A. Impact of SARS-CoV-2 on saliva: TNF-, IL-6, IL-10, lactoferrin, lysozyme, IgG, IgA, and IgM. J Oral Biosci (2022) 64(1):108–13. doi: 10.1016/j.job.2022.01.007 PMC8788095 35091065

[75] Waltz E. How nasal-spray vaccines could change the pandemic. Nature (2022) 609(7926):240–2. doi: 10.1038/d41586-022-02824-3 36068305

[76] Tang J Zeng C Cox TM Li C Son YM Cheon IS. Respiratory mucosal immunity against SARS-CoV-2 after mRNA vaccination. Sci Immunol (2022) 7(76):eadd4853. doi: 10.1126/sciimmunol.add4853 35857583 PMC9348751

[77] Li M Ye ZW Tang K Guo L Bi W Zhang Y. Enhanced trimeric ACE2 exhibits potent prophylactic and therapeutic efficacy against the SARS-CoV-2 delta and omicron variants. vivo. Cell Res (2022) 32(6):589–92. doi: 10.1038/s41422-022-00656-4 PMC9007249 35418217

[78] Brevini T Maes M Webb GJ John BV Fuchs CD Buescher G. FXR inhibition may protect from SARS-CoV-2 infection by reducing ACE2. Nature (2022). doi: 10.1038/s41586-022-05594-0 PMC9977684 36470304

[79] Ghosh S Kumar M Santiana M Mishra A Zhang M Labayo H. Enteric viruses replicate in salivary glands and infect through saliva. Nature (2022) 607(7918):345–50. doi: 10.1038/s41586-022-04895-8 PMC9243862 35768512

[80] Azzi L Dalla Gasperina D Veronesi G Shallak M Ietto G Iovino D. Mucosal immune response in BNT162b2 COVID-19 vaccine recipients. EBioMedicine (2022) 75:103788. doi: 10.1016/j.ebiom.2021.103788 34954658 PMC8718969

[81] Jin L Guo X Shen C Hao X Sun P Li P. Salivary factor LTRIN from aedes aegypti facilitates the transmission of zika virus by interfering with the lymphotoxin-β receptor. Nat Immunol (2018) 19(4):342–53. doi: 10.1038/s41590-018-0063-9 29507355

[82] Hopp CS Sinnis P. The innate and adaptive response to mosquito saliva and plasmodium sporozoites in the skin. Ann N Y Acad Sci (2015) 1342(1):37–43. doi: 10.1111/nyas.12661 25694058 PMC4405444

[83] Ogle OE. Salivary gland diseases. Dent Clin North Am (2020) 64(1):87–104. doi: 10.1016/j.cden.2019.08.007 31735235

[84] Murakami S Mealey BL Mariotti A Chapple ILC. Dental plaque-induced gingival conditions. J Clin Periodontol (2018) 45(Suppl 20):S17–27. doi: 10.1111/jcpe.12937 29926503

[85] Holmstrup P Plemons J Meyle J. Non-plaque-induced gingival diseases. J Clin Periodontol (2018) 45 Suppl 20:S28–43. doi: 10.1111/jcpe.12938 29926497

[86] K inane DF Stathopoulou PG Papapanou PN. Periodontal diseases. Nat Rev Dis Primers (2017) 3:17038. doi: 10.1038/nrdp.2017.38 28805207

[87] Brito-Zeron P Baldini C Bootsma H Bowman SJ Jonsson R Mariette X. Sjogren syndrome. Nat Rev Dis Primers (2016) 2:16047. doi: 10.1038/nrdp.2016.47 27383445

[88] Hwang SH Woo JS Moon J Yang S Park JS Lee J. IL-17 and CCR9^+^alpha4beta7^-^ Th17 cells promote salivary gland inflammation, dysfunction, and cell death in sjogren's syndrome. Front Immunol (2021) 12:721453. doi: 10.3389/fimmu.2021.721453 34539657 PMC8440850

[89] Skutnik-Radziszewska A Maciejczyk M Flisiak I Kolodziej JKU Kotowska-Rodziewicz A Klimiuk A. Enhanced inflammation and nitrosative stress in the saliva and plasma of patients with plaque psoriasis. J Clin Med (2020) 9(3):745. doi: 10.3390/jcm9030745 32164227 PMC7141316

[90] Skutnik-Radziszewska A Maciejczyk M Fejfer K Krahel J Flisiak I Kolodziej U. Salivary antioxidants and oxidative stress in psoriatic patients: can salivary total oxidant status and oxidative status index be a plaque psoriasis biomarker? Oxid Med Cell Longev (2020) 2020:9086024. doi: 10.1155/2020/9086024 31998446 PMC6964728

[91] Senapati S Banerjee P Bhagavatula S Kushwaha PP Kumar S. Contributions of human ACE2 and TMPRSS2 in determining host–pathogen interaction of COVID-19. J Genet (2021) 100(1):12. doi: 10.1007/s12041-021-01262-w 33707363 PMC7904510

[92] Datta PK Liu F Fischer T Rappaport J Qin X. SARS-CoV-2 pandemic and research gaps: Understanding SARS-CoV-2 interaction with the ACE2 receptor and implications for therapy. Theranostics (2020) 10(16):7448–64. doi: 10.7150/thno.48076 PMC7330865 32642005

[93] Zupin L Pascolo L Crovella S. Is FURIN gene expression in salivary glands related to SARS-CoV-2 infectivity through saliva? J Clin Pathol (2021) 74(4):209–11. doi: 10.1136/jclinpath-2020-206788 32660959

[94] Salas Orozco MF Nino-Martinez N Martinez-Castanon GA Patino Marin N Samano Valencia C Dipp Velazquez FA. Presence of SARS-CoV-2 and its entry factors in oral tissues and cells: a systematic review. Medicina (Kaunas) (2021) 57(6):523. doi: 10.3390/medicina57060523 34070998 PMC8224617

[95] Sakaguchi W Kubota N Shimizu T Saruta J Fuchida S Kawata A. Existence of SARS-CoV-2 entry molecules in the oral cavity. Int J Mol Sci (2020) 21(17):6000. doi: 10.3390/ijms21176000 32825469 PMC7503451

[96] Zhu F Zhong Y Ji H Ge R Guo L Song H. ACE2 and TMPRSS2 in human saliva can adsorb to the oral mucosal epithelium. J Anat (2022) 240(2):398–409. doi: 10.1111/joa.13560 34590312 PMC8662096

[97] Matuck BF Dolhnikoff M Duarte-Neto AN Maia G Gomes SC Sendyk DI. Salivary glands are a target for SARS-CoV-2: a source for saliva contamination. J Pathol (2021) 254(3):239–43. doi: 10.1002/path.5679 PMC8250228 33834497

[98] Scialo F Daniele A Amato F Pastore L Matera MG Cazzola M. ACE2: the major cell entry receptor for SARS-CoV-2. Lung (2020) 198(6):867–77. doi: 10.1007/s00408-020-00408-4 PMC7653219 33170317

[99] Jain A Lamperti M Lobo FA. Renin-Angiotensin-Aldosterone system imbalance and altered aquaporin activity: a new perspective for COVID-19-associated xerostomia. Ear Nose Throat J (2021), 1455613211030348. doi: 10.1177/01455613211030348 34233506

[100] Tsuchiya H. Oral symptoms associated with COVID-19 and their pathogenic mechanisms: a literature review. Dent J (Basel) (2021) 9(3):32. doi: 10.3390/dj9030032 33799583 PMC7999671

[101] Patel SK Juno JA Lee WS Wragg KM Hogarth PM Kent SJ. Plasma ACE2 activity is persistently elevated following SARS-CoV-2 infection: implications for COVID-19 pathogenesis and consequences. Eur Respir J (2021) 57(5):2003730. doi: 10.1183/13993003.03730-2020 33479113 PMC7830336

[102] Wang C Wu H Ding X Ji H Jiao P Song H. Does infection of 2019 novel coronavirus cause acute and/or chronic sialadenitis? Med Hypotheses (2020) 140:109789. doi: 10.1016/j.mehy.2020.109789 32361098 PMC7194735

[103] Stuck BA Frey S Freiburg C Hormann K Zahnert T Hummel T. Chemosensory event-related potentials in relation to side of stimulation, age, sex, and stimulus concentration. Clin Neurophysiol (2006) 117(6):1367–75. doi: 10.1016/j.clinph.2006.03.004 16651024

[104] Wolff A Joshi RK Ekstrom J Aframian D Pedersen AM Proctor G. A guide to medications inducing salivary gland dysfunction, xerostomia, and subjective sialorrhea: a systematic review sponsored by the world workshop on oral medicine VI. Drugs R D (2017) 17(1):1–28. doi: 10.1007/s40268-016-0153-9 27853957 PMC5318321

[105] Aliko A Wolff A Dawes C Aframian D Proctor G Ekstrom J. World workshop on oral medicine VI: clinical implications of medication-induced salivary gland dysfunction. Oral Surg Oral Med Oral Pathol Oral Radiol (2015) 120(2):185–206. doi: 10.1016/j.oooo.2014.10.027 25861957

[106] Jensen SB Vissink A Limesand KH Reyland ME. Salivary gland hypofunction and xerostomia in head and neck radiation patients. J Natl Cancer Inst Monogr (2019) 2019(53):lgz016. doi: 10.1093/jncimonographs/lgz016 31425600

[107] Raz E Saba L Hagiwara M Hygino de Cruz LC Jr. Som PM Fatterpekar GM. Parotid gland atrophy in patients with chronic trigeminal nerve denervation. AJNR Am J Neuroradiol (2013) 34(4):860–3. doi: 10.3174/ajnr.A3290 PMC7964499 23042921

[108] Li X Azlina A Karabasil MR Purwanti N Hasegawa T Yao C. Degradation of submandibular gland AQP5 by parasympathetic denervation of chorda tympani and its recovery by cevimeline, an M3 muscarinic receptor agonist. Am J Physiol Gastrointest Liver Physiol (2008) 295(1):G112–G23. doi: 10.1152/ajpgi.00359.2007 18450949

[109] Kent M Song RB Glass EN de Lahunta A. A salivation abnormality with trigeminal nerve dysfunction in dogs. J Vet Dent (2019) 36(1):8–16. doi: 10.1177/0898756419846607 31138049

[110] Mauri-Obradors E Estrugo-Devesa A Jane-Salas E Vinas M Lopez-Lopez J. Oral manifestations of diabetes mellitus. A systematic review. Med Oral Patol Oral Cir Bucal (2017) 22(5):e586-e94. doi: 10.4317/medoral.21655 28809366 PMC5694181

[111] Xiang RL Huang Y Zhang Y Cong X Zhang ZJ Wu LL. Type 2 diabetes-induced hyposalivation of the submandibular gland through PINK1/Parkin-mediated mitophagy. J Cell Physiol (2020) 235(1):232–44. doi: 10.1002/jcp.28962 PMC6851669 31190343

[112] Albuquerque DF de Souza Tolentino E Amado FM Arakawa C Chinellato LE. Evaluation of halitosis and sialometry in patients submitted to head and neck radiotherapy. Med Oral Patol Oral Cir Bucal (2010) 15(6):e850–4. doi: 10.4317/medoral.15.e850 20383099

[113] Serrano J Lopez-Pintor RM Gonzalez-Serrano J Fernandez-Castro M Casanas E Hernandez G. Oral lesions in sjogren's syndrome: A systematic review. Med Oral Patol Oral Cir Bucal (2018) 23(4):e391–400. doi: 10.4317/medoral.22286 PMC6051685 29924754

[114] Crincoli V Di Comite M Guerrieri M Rotolo RP Limongelli L Tempesta A. Orofacial manifestations and temporomandibular disorders of sjogren syndrome: an observational study. Int J Med Sci (2018) 15(5):475–83. doi: 10.7150/ijms.23044 PMC5859770 29559836

[115] Buranarom N Komin O Matangkasombut O. Hyposalivation, oral health, and candida colonization in independent dentate elders. PloS One (2020) 15(11):e0242832. doi: 10.1371/journal.pone.0242832 33237956 PMC7688165

[116] Nadig SD Ashwathappa DT Manjunath M Krishna S Annaji AG Shivaprakash PK. A relationship between salivary flow rates and candida counts in patients with xerostomia. J Oral Maxillofac Pathol (2017) 21(2):316. doi: 10.4103/jomfp.JOMFP_231_16 28932047 PMC5596688

[117] Flink H Tegelberg A Arnetz JE Birkhed D. Self-reported oral and general health related to xerostomia, hyposalivation, and quality of life among caries active younger adults. Acta Odontol Scand (2020) 78(3):229–35. doi: 10.1080/00016357.2019.1690677 31729277

[118] Amezaga J Alfaro B Rios Y Larraioz A Ugartemendia G Urruticoechea A. Assessing taste and smell alterations in cancer patients undergoing chemotherapy according to treatment. Support Care Cancer (2018) 26(12):4077–86. doi: 10.1007/s00520-018-4277-z 29855774

[119] Munoz-GonzalezCVandenberghe-DescampsMFeronGCanonFLaboureHSulmont-RosseC. Association between salivary hypofunction and food consumption in the elderlies. a systematic literature review. J Nutr Health Aging (2018) 22(3):407–19. doi: 10.1007/s12603-017-0960-x 29484355

[120] Munoz-Gonzalez C Feron G Canon F. Main effects of human saliva on flavour perception and the potential contribution to food consumption. Proc Nutr Soc (2018) 77(4):423–31. doi: 10.1017/S0029665118000113 29661256

[121] Iwasaki M Yoshihara A Ito K Sato M Minagawa K Muramatsu K. Hyposalivation and dietary nutrient intake among community-based older Japanese. Geriatr Gerontol Int (2016) 16(4):500–7. doi: 10.1111/ggi.12500 25952943

[122] Cui Y Li J Li L Zhao Q Chen S Xia L. Prevalence, correlates, and impact of sleep disturbance in Chinese patients with primary sjögren’s syndrome. Int J Rheumatic Dis (2019) 23(3):367–73. doi: 10.1111/1756-185x.13678 31763772

[123] Grover SS Rhodus NL. Xerostomia and depression. Northwest Dent (2016) 95(3):29, 31, 3–5. 27476240

[124] Farshidfar N Hamedani S. Hyposalivation as a potential risk for SARS-CoV-2 infection: Inhibitory role of saliva. Oral Dis (2021) 27(Suppl 3):750–1. doi: 10.1111/odi.13375 PMC7267261 32348636

[125] Iwabuchi H Fujibayashi T Yamane GY Imai H Nakao H. Relationship between hyposalivation and acute respiratory infection in dental outpatients. Gerontology (2012) 58(3):205–11. doi: 10.1159/000333147 22104982

[126] Arias-Bujanda N Regueira-Iglesias A Balsa-Castro C Nibali L Donos N Tomas I. Accuracy of single molecular biomarkers in gingival crevicular fluid for the diagnosis of periodontitis: A systematic review and meta-analysis. J Clin Periodontol (2019) 46(12):1166–82. doi: 10.1111/jcpe.13188 31444912

[127] Korte DL Kinney J. Personalized medicine: an update of salivary biomarkers for periodontal diseases. Periodontol 2000 (2016) 70(1):26–37. doi: 10.1111/prd.12103 26662480

[128] AlMoharib HS AlMubarak A AlRowis R Geevarghese A Preethanath RS Anil S. Oral fluid based biomarkers in periodontal disease: part 1. Saliva. J Int Oral Health (2014) 6(4):95–103. 25214743 PMC4148585

[129] Lahdentausta LSJ Paju S Mantyla P Buhlin K Tervahartiala T Pietiainen M. Saliva and serum biomarkers in periodontitis and coronary artery disease. J Clin Periodontol (2018) 45(9):1045–55. doi: 10.1111/jcpe.12976 29972696

[130] Lira-Junior R Holmström SB Clark R Zwicker S Majster M Johannsen G. S100A12 expression is modulated during monocyte differentiation and reflects periodontitis severity. Front Immunol (2020) 11:86. doi: 10.3389/fimmu.2020.00086 32082330 PMC7005221

[131] Grande MA Belstrom D Damgaard C Holmstrup P Thangaraj SS Nielsen CH. Complement split product C3c in saliva as biomarker for periodontitis and response to periodontal treatment. J Periodontal Res (2021) 56(1):27–33. doi: 10.1111/jre.12788 32681659 PMC7891408

[132] Jung JY Kim JW Kim HA Suh CH. Salivary biomarkers in patients with sjogren's syndrome-a systematic review. Int J Mol Sci (2021) 22(23):12903. doi: 10.3390/ijms222312903 34884709 PMC8657642

[133] Hu S Wang J Meijer J Ieong S Xie Y Yu T. Salivary proteomic and genomic biomarkers for primary sjogren's syndrome. Arthritis Rheum (2007) 56(11):3588–600. doi: 10.1002/art.22954 PMC2856841 17968930

[134] Odeh ND Babkair H Abu-Hammad S Borzangy S Abu-Hammad A Abu-Hammad O. COVID-19: present and future challenges for dental practice. Int J Environ Res Public Health (2020) 17(9):3151. doi: 10.3390/ijerph17093151 32366034 PMC7246705

[135] Xu R Cui B Duan X Zhang P Zhou X Yuan Q. Saliva: potential diagnostic value and transmission of 2019-nCoV. Int J Oral Sci (2020) 12(1):11. doi: 10.1038/s41368-020-0080-z 32300101 PMC7162686

[136] To KK Tsang OT Yip CC Chan KH Wu TC Chan JM. Consistent detection of 2019 novel coronavirus in saliva. Clin Infect Dis (2020) 71(15):841–3. doi: 10.1093/cid/ciaa149 PMC7108139 32047895

[137] To KK Tsang OT Leung WS Tam AR Wu TC Lung DC. Temporal profiles of viral load in posterior oropharyngeal saliva samples and serum antibody responses during infection by SARS-CoV-2: an observational cohort study. Lancet Infect Dis (2020) 20(5):565–74. doi: 10.1016/S1473-3099(20)30196-1 PMC7158907 32213337

[138] Zhang W Du RH Li B Zheng XS Yang XL Hu B. Molecular and serological investigation of 2019-nCoV infected patients: implication of multiple shedding routes. Emerg Microbes Infect (2020) 9(1):386–9. doi: 10.1080/22221751.2020.1729071 PMC7048229 32065057

[139] Pundir M Papagerakis S De Rosa MC Chronis N Kurabayashi K Abdulmawjood S. Emerging biotechnologies for evaluating disruption of stress, sleep, and circadian rhythm mechanism using aptamer-based detection of salivary biomarkers. Biotechnol Adv (2022) 59:107961. doi: 10.1016/j.biotechadv.2022.107961 35427723

[140] de Almeida EA Di Mascio P Harumi T Spence DW Moscovitch A Hardeland R. Measurement of melatonin in body fluids: standards, protocols and procedures. Childs Nerv Syst (2011) 27(6):879–91. doi: 10.1007/s00381-010-1278-8 PMC3128751 21104186

[141] Inder WJ Dimeski G Russell A. Measurement of salivary cortisol in 2012 - laboratory techniques and clinical indications. Clin Endocrinol (Oxf) (2012) 77(5):645–51. doi: 10.1111/j.1365-2265.2012.04508.x 22812714

[142] Jantaratnotai N Rungnapapaisarn K Ratanachamnong P Pachimsawat P. Comparison of salivary cortisol, amylase, and chromogranin a diurnal profiles in healthy volunteers. Arch Oral Biol (2022) 142:105516. doi: 10.1016/j.archoralbio.2022.105516 35952574

[143] Ceccato F Boscaro M. Cushing's syndrome: screening and diagnosis. High Blood Press Cardiovasc Prev (2016) 23(3):209–15. doi: 10.1007/s40292-016-0153-4 27160717

[144] Ceccato F Marcelli G Martino M Concettoni C Brugia M Trementino L. The diagnostic accuracy of increased late night salivary cortisol for cushing's syndrome: a real-life prospective study. J Endocrinol Invest (2019) 42(3):327–35. doi: 10.1007/s40618-018-0921-1 29987756

[145] Majster M Lira-Junior R Hoog CM Almer S Bostrom EA. Salivary and serum inflammatory profiles reflect different aspects of inflammatory bowel disease activity. Inflammation Bowel Dis (2020) 26(10):1588–96. doi: 10.1093/ibd/izaa190 PMC7500518 32725166

[146] Borsting T Venkatraman V Fagerhaug TN Skeie MS Stafne SN Feuerherm AJ. Systematic assessment of salivary inflammatory markers and dental caries in children: an exploratory study. Acta Odontol Scand (2022) 80(5):338–45. doi: 10.1080/00016357.2021.2011400 34875210

[147] Lisa Cheng YS Jordan L Gorugantula LM Schneiderman E Chen HS Rees T. Salivary interleukin-6 and -8 in patients with oral cancer and patients with chronic oral inflammatory diseases. J Periodontol (2014) 85(7):956–65. doi: 10.1902/jop.2013.130320 24147842

[148] Panneer Selvam N Sadaksharam J. Salivary interleukin-6 in the detection of oral cancer and precancer. Asia Pac J Clin Oncol (2015) 11(3):236–41. doi: 10.1111/ajco.12330 25560781

[149] Kaur J Jacobs R. Proinflammatory cytokine levels in oral lichen planus, oral leukoplakia, and oral submucous fibrosis. J Kor Assoc Oral Max (2015) 41(4):171–5. doi: 10.5125/jkaoms.2015.41.4.171 PMC4558184 26339574

[150] Mehterov N Vladimirov B Sacconi A Pulito C Rucinski M Blandino G. Salivary miR-30c-5p as potential biomarker for detection of oral squamous cell carcinoma. Biomedicines (2021) 9(9):1079. doi: 10.3390/biomedicines9091079 34572265 PMC8465705

[151] McDew-White M Lee E Alvarez X Sestak K Ling BJ Byrareddy SN. Cannabinoid control of gingival immune activation in chronically SIV-infected rhesus macaques involves modulation of the indoleamine-2,3-dioxygenase-1 pathway and salivary microbiome. EBioMedicine (2022) 75:103769. doi: 10.1016/j.ebiom.2021.103769 34954656 PMC8715300

[152] Maciejczyk M Szulimowska J Skutnik A Taranta-Janusz K Wasilewska A Wisniewska N. Salivary biomarkers of oxidative stress in children with chronic kidney disease. J Clin Med (2018) 7(8):209. doi: 10.3390/jcm7080209 30103431 PMC6111793

[153] Maciejczyk M Szulimowska J Taranta-Janusz K Werbel K Wasilewska A Zalewska A. Salivary FRAP as a marker of chronic kidney disease progression in children. Antioxidants (Basel) (2019) 8(9):409. doi: 10.3390/antiox8090409 31540400 PMC6769502

